# Nitrogen removal performance and the biocenosis with microalgae consortium Nitrosifying and anammox bacteria in an upflow reactor

**DOI:** 10.1016/j.heliyon.2024.e34794

**Published:** 2024-07-18

**Authors:** Xiangyin Liu, Jiannv Chen, Tiansheng Lu, Yujie Qin

**Affiliations:** a School of Environment and Energy, South China University of Technology, Guangzhou, China; b The Key Lab of Pollution Control and Ecosystem Restoration in Industry Clusters, Ministry of Education, South China University of Technology, Guangzhou Higher Education Mega Centre, Guangzhou, 510006, China

**Keywords:** Microalgae, Partial nitrification, Anammox, Algal-bacterial consortium

## Abstract

This study introduced an innovative pathway utilizing an algal anaerobic ammonium oxidation (ALGAMMOX) system to treat ammonium wastewater. Lake bottom sludge and anammox sludge were used to cultivate functional microorganisms and microalgae for nitrogen removal in an upflow reactor made of transparent materials. The results showed that the ALGAMMOX system achieved 87.40 % nitrogen removal when the influent NH_4_^+^-N concentration was 100 mg-N/L. Further analysis showed that anammox bacteria *Candidatus Brocadia* (8.87 %) and nitrosobacteria *Nitrosomonas* (3.74 %) were crucial contributors, playing essential roles in nitrogen removal. The 16S rRNA gene showed that the anammox bacteria in the sludge transitioned from *Candidatus Kuenenia* to *Candidatus Brocadia*. The 18S rRNA gene revealed that *Chlamydomonas, Bacillariaceae* and *Pinnularia* were the dominant microalgae in the system at a relative abundance of 7.99 %, 3.64 % and 3.14 %, respectively. This novel approach provides a theoretical foundation for ammonium wastewater treatment.

## Highlights

• The ALGAMMOX system was successfully established inoculating lake sediment and anammox sludge.
• *Chlamydomonas*, *Bacillariaceae* and *Pinnularia* became the dominant algae in ALGAMMOX system.
• The highest TN removal efficiency was 87.40 % in the ALGAMMOX system.
• The anammox bacteria transitioned from *Candidatus Kuenenia* to *Candidatus Brocadia.*

## 1 Introduction

Partial nitrification-anammox (PN/A) is considered an efficient and energy-saving biological nitrogen removal technology due to its low oxygen demand and lack of need for additional organic carbon sources [1]. In recent years, biological nitrogen removal using PN/A combined with microalgae has attracted significant attention because it is more energy-saving and environmentally friendly [2,3]. Adding microalgae to the PN/A system is important. Microalgae provide oxygen through photosynthesis in the PN/A system, and they also promote nitrogen removal and provide a denitrification substrate [4,5]. The completely autotrophic algal anaerobic ammonium oxidation (ALGAMMOX) combines microalgae, ammonia oxidizing bacteria (AOB), and anaerobic ammonium oxidation bacteria (AnAOB) to achieve biological nitrogen removal [6]. Microalgae utilize carbon dioxide, water, and inorganic matter produced by bacterial decomposition for photosynthesis, producing oxygen during the photoperiod. The AOB use the oxygen provided by microalgae to convert ammonium to nitrite. The AnAOB further convert ammonium and nitrite to nitrogen (N_2_) and a small amount of nitrate during the dark cycle.

Research on algae-intensified PN/A systems is mostly in the exploratory stage, focusing on various operational parameters such as light intensity, photoperiod, and microalgal species to enhance their feasibility [7]. Light plays a pivotal role in algae-bacteria symbiotic systems [8]. Adjusting light conditions can facilitate the startup of the ALGAMMOX biofilm system and achieve effective nitrogen removal [2]. Ultraviolet-A has been demonstrated to effectively reduce the abundance of nitrite-oxidizing bacteria (NOB) without affecting AOB [9]. Stable biological nitrogen removal was successfully attained in a photobioreactor with an algal-bacteria consortium under alternating 12-h light and 12-h dark conditions [10]. It is noteworthy that natural light may be more beneficial for algal growth compared to artificial light [11]. Natural sunlight provides a full spectrum of light wavelengths that artificial lighting systems often cannot replicate accurately. In addition, utilizing natural light reduces the dependency on artificial lighting, which can be energy-intensive and costly. This makes the ALGAMMOX system more sustainable and economically viable. However, limited research has been conducted on the regulation of ALGAMMOX system operation under natural light conditions. This is meaningful for the further development of algal-bacterial symbiotic systems.

The compatibility of microalgae with wastewater requires consideration [12]. A previous study achieved high pollutant removal efficiency using lake water enriched with algae and bacteria [13]. Lake bottom sludge contains a diverse range of microalgae and bacterial populations with nitrogen removal capabilities, making it an excellent choice for initial sludge.

In this study, two objectives were pursued: i) To initiate and optimize an ALGAMMOX system using lake bottom sludge and anammox sludge indoors under natural light, focusing on maximizing the nitrogen removal efficiency by combining microalgae, AOB, and AnAOB. ii) To investigate the microbial community structure of the system, including species composition, functional diversity, and roles in the nitrogen removal process. This study may provide new insights for ALGAMMOX and serve as a theoretical basis for the further development of the ALGAMMOX process.

## 2 Materials and methods

### 2.1 Sludge and simulated wastewater

The initial inoculated sludge was the lake bottom sludge from the central lake of Guangzhou University City (Guangzhou, China). Rhizomes and large gravel particles were sieved through a 2-mm sieve before inoculation. Anammox sludge, capable of consistently removing ammonium and nitrite, was inoculated into the reactor after it had been operational for 20 days. The relative abundance of AnAOB (*Candidatus Kuenenia*) in the anammox sludge was 4.83 %.

Ammonium chloride and sodium nitrite were used to supply NH_4_^+^-N and NO_2_^−^-N, respectively. The influent wastewater was simulated using synthetic wastewater during the study. The chemicals used to synthesize the wastewater were sourced from Tianjin Damao Chemical Reagent Factory and were of analytical grade purity. The nutrient solution components were NaHCO_3_ (1000 mg/L), CaCl_2_–2H_2_O (180 mg/L), KH_2_PO_4_ (27 mg/L), and MgSO_4_–7H_2_O (300 mg/L). Trace element solutions I and II were prepared based on our previous research [14]. HCl (1.0 mol/L) and NaOH (1.0 mol/L) were used to maintain the pH of the influent water at 7.95 ± 0.40.

### 2.2 Experimental setup and reactor operation

#### 2.2.1 Experimental setup

An upflow reactor made of polymethyl methacrylate was designed to facilitate light penetration and reach the biomass. The reactor consisted of a 4 L cylindrical tank equipped with an automatic feeding system. The reaction zone was cylindrical, with an inner diameter of 10.40 cm, an outer diameter of 11.00 cm, and a height of 50.00 cm. It operated continuously at flow rates specified for each experimental phase (0.90 mL/min, 1.40 mL/min, 2.80 mL/min). It was placed next to a window to absorb natural light, with no additional light source required. The reactor relied solely on microalgae for oxygen production without additional aeration. The pH was stabilized at 7.95 ± 0.40 throughout the experiment.

#### 2.2.2 Reactor operation

The experimental process was divided into four stages in the same reactor, as shown in Table 1. The process of acclimation and growth of microalgae was included in stage Ⅰ (1–19 days). Stage Ⅱ (20–113 days) gradually built up a fully autotrophic ALGAMMOX system by regulating the NH_4_^+^-N and NO_2_^−^-N concentrations in the influent. Stage Ⅲ (114–240 days) focused on reducing the Hydraulic Retention Time (HRT). Reducing HRT lowers the area requirements of the system, which is important for the application of the ALGAMMOX process. In stage Ⅳ (241–289 days), the reactor performance was further optimized to build a mature and stable ALGAMMOX system by further reducing the influent concentration of NO_2_^−^-N.

**Table 1:** The operating conditions of up-flow reactor during four experimental stages.

Stage	Time(d)	HRT(h)	Influent concentration (mg-N/L)		Molar ratio NO_2_^−^-N/NH_4_^+^-N
			NH_4_^+^-N	NO_2_^−^-N	
Ⅰ	1–19	72	40	0	0
Ⅱ	20–30	72	40	52	1.30
	31–62	72	40	26	0.65
	63–113	72	100	55	0.55
Ⅲ	114–126	72	100	55	0.55
	127–178	48	100	55	0.55
	179–240	24	100	55	0.55
Ⅳ	241–254	48	100	55	0.55
	255–268	48	100	45	0.45
	269–274	48	100	40	0.40
	275–289	48	100	35	0.35

### 2.3 Analytical methods

Concentrations of NH_4_^+^-N, NO_2_^−^-N, and NO_3_^−^-N were determined every two days by spectrophotometry. Before analysis, wastewater samples were passed through 0.45 μm filter paper. Each sample was tested three times per analysis to obtain an average value. The temperature was measured using a digital portable thermometer, and a handheld digital pH meter was used to monitor pH.

### 2.4 Microbial community analysis

Microorganisms and the algal-bacterial consortium in the sludge were observed using a light microscope. Initial lake bottom sludge sample L_0_ and anaerobic ammonia-oxidizing sludge sample A_0_, as well as sludge sample L_289_ collected on day 289 of the experiment, were stored at −20 °C for subsequent DNA extraction. The total genome was isolated using the PowerSoil DNA Isolation Kit (Mo Bio Laboratories, USA) according to the manufacturer's instructions and was checked for quality and quantity using a Nanodrop spectrophotometer (ND-1000, NanoDrop Technologies, USA). Polymerase chain reaction (PCR) and high-throughput sequencing were performed as described in our previous study [15].

## 3 Results and discussion

### 3.1 Nitrogen removal performance

**Fig. 1:**
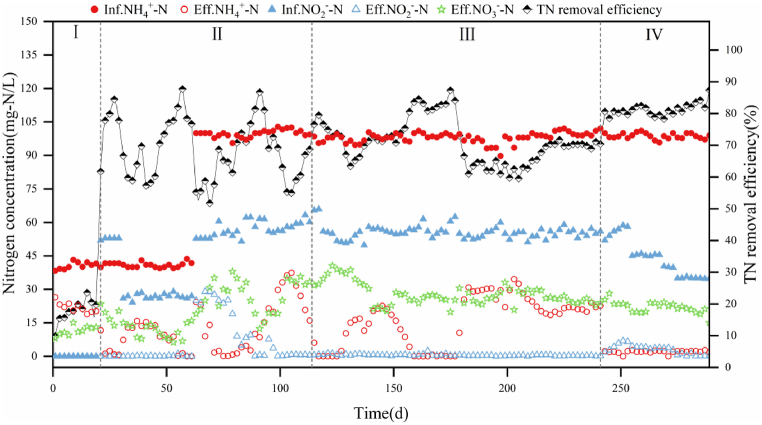
Nitrogen removal performance in the reactor.

The nitrogen removal capacity of the reactor during the four stages was illustrated in Fig. 1. During stage Ⅰ (from day 1 to day 19), the HRT was set at 72 h. The influent concentration of NH_4_^+^-N was 40 mg-N/L for the first 20 days following the addition of lake bottom sludge. The NH_4_^+^-N removal efficiency reached 51.29 % on the 19th day, demonstrating the considerable nitrogen removal capacity of the lake bottom sludge. However, the total nitrogen (TN) removal efficiency was only 19.78 % due to NO_3_^−^-N production. Analysis of the 16S rRNA gene revealed the presence of NOB in the original lake bottom sludge. In the initial stages of reactor startup, the activity of NOB was high, leading to significant complete nitrification reactions within the system. Continued growth of microalgae was evident, as observed by their attachment to the reactor wall during this period.

In the stage Ⅱ (from day 20 to day 113), anammox sludge was introduced on day 20. From day 21 to day 30, NH_4_^+^-N and NO_2_^−^-N were added to the influent at concentrations of 40 mg-N/L and 52 mg-N/L, respectively, with a NO_2_^−^-N/NH_4_^+^-N molar ratio of 1.30, creating an adapted environment for the anammox bacteria. The AnAOB adapted well to the microaerobic environment, resulting in nearly complete elimination of NH_4_^+^-N and NO_2_^−^-N. On the 31st day, the influent concentration of NH_4_^+^-N remained at 40 mg-N/L, and the influent NO_2_^−^-N concentration dropped from 52 mg-N/L to 26 mg-N/L. The aim was to enrich the AOB. The NO_3_^−^-N in the effluent gradually decreased from 20.37 mg-N/L to 7.82 mg-N/L between days 29 and 39. The system was deficient in the electron acceptor (NO_2_^−^-N) required for AnAOB due to the sudden halving of the artificially supplemented NO_2_^−^-N. The substrate concentration in the reactor became more favorable for AOB growth. From day 31 to day 37, the NH_4_^+^-N in the effluent increased from 0.63 mg-N/L to 15.83 mg-N/L, while NO_2_^−^-N was barely detectable in the effluent. NH_4_^+^-N removal began to increase on day 37. On the 61st day, the NH_4_^+^-N removal efficiency reached 100 %, and the TN removal efficiency reached 76.74 %. The ALGAMMOX system was successfully started up. On the 63rd day, the influent NH_4_^+^-N concentration was elevated to a typical level of 100 mg-N/L, with an influent NO_2_^−^-N concentration of 55 mg-N/L for the reactor stabilization. The NH_4_^+^-N concentration in the effluent initially firstly increased 24.34 mg-N/L from 0 mg-N/L, and then began to decrease after 5 days of acclimatization. The NO_2_^−^-N was almost undetectable in the effluent water, which indicated that the AnAOB were still maintained high activity. On the 87th day, the NH_4_^+^-N removal efficiency reached 97.26 %, and the TN removal efficiency reached 76.78 %. A drop in temperature caused the system to go out of control between day 89 and day 105, with the temperature decreasing from 24.5 °C to 16.9 °C. On the 95th day, the temperature dropped to a minimum of 16.9 °C. By the 105th day, the TN removal efficiency had decreased to a minimum of 55.02 %. After the temperature rebounded, the reactor's nitrogen removal performance gradually recovered. By the 113th day, the NH_4_^+^-N removal efficiency had recovered to 83.87 %, and the TN removal efficiency had recovered to 68.74 %. After this low-temperature phase, the effluent concentration of NO_3_^−^-N remained higher than the theoretical value, possibly because NOB are more resistant to low temperatures than AOB [16].

**Fig. 2:**
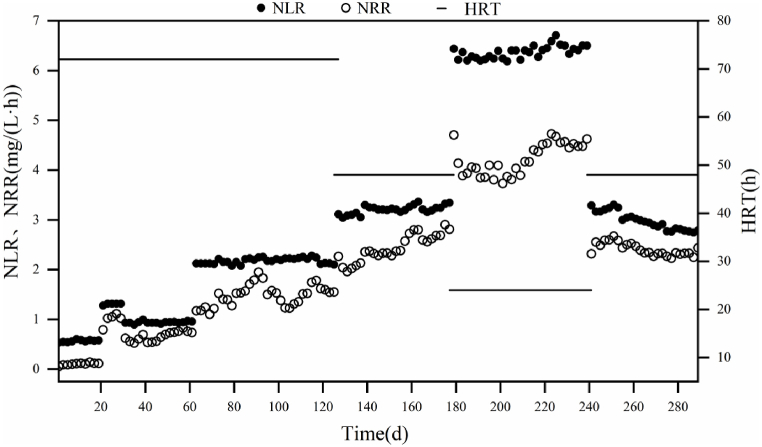
HRT, NLR and NRR of the reactor operation process.

In the stage Ⅲ (from day 114 to day 240), the system was operated with an HRT of 72 h On days 114–126 (Fig. 2). During this period, the reactor NH_4_^+^-N removal efficiency reached 100 %. On the 127th day, the HRT was shortened to 48 h. However, as the HRT was reduced, the effluent NH_4_^+^-N concentration increased from nearly 0 mg-N/L to 22.63 mg-N/L, and the TN removal efficiency gradually declined from 79.48 % to 63.48 %. From day 145 to day 175, the NH_4_^+^-N removal efficiency gradually increased to 100 % and the TN removal efficiency gradually increased from 71.22 % to 87.31 %. The nitrogen removal rate (NRR) increased from 1.96 mg L^−1^ h^−1^ to 2.88 mg L^−1^ h^−1^ on days 130–175. On the 179th day, the HRT was further reduced to 24 h. A significant increase in NRR was observed after further shortening the HRT to 24 h from day 176 to day 223. On the 219th day, NH_4_^+^-N removal efficiency increased to 81.09 % and TN removal efficiency was 70.42 %. However, unlike previously, the NH_4_^+^-N removal efficiency did not return to its previous high level. From day 1 to day 223, the NRR showed an upward trend as shown in Fig. 2. On the 223rd day, the nitrogen loading rate (NLR) was 6.59 mg L^−1^ h^−1^ and the NRR reached a maximum of 4.73 mg L^−1^ h^−1^. The experimental findings indicated that an HRT of 24 h is beneficial for achieving optimal nitrogen removal performance in continuous upflow reactors.

In Stage IV (from day 241 to day 289), to maintain high removal efficiency, the HRT was set to 48 h. Additional nitrite added was gradually reduced starting on day 255, with each reduction limited to 10 mg-N/L to ensure system stability. From days 255–268, NH_4_^+^-N removal efficiency remained above 95.78 %, and TN removal efficiency remained above 78.80 % with a NO_2_^−^-N influent concentration of 45 mg-N/L. From days 269–274, with the influent NO_2_^−^-N concentration reduced to 40 mg-N/L, NH_4_^+^-N removal efficiency was above 97.67 %, and TN removal efficiency was above 78.28 %. From days 275–289, the NO_2_^−^-N concentration was further reduced to 35 mg-N/L, and by day 289, the system achieved a TN removal efficiency of 87.40 %.

### 3.2 Prokaryotic microbial communities

#### 3.2.1 Alpha diversity analysis

Table 2 presented the Alpha diversity indices of the microbial community. 16sL_0_ and 16sL_289_ represented the sludge samples at the beginning and end of the experiment, respectively, and 16sA_0_ represented the initial inoculum of anaerobic sludge. According to the ACE index and Chao1 indices, the species richness of microorganisms in the reactor was lower than that of the initially inoculated lake bottom sludge. Similarly, both Shannon and Simpson indices indicated that the microbial community diversity in the reactor was reduced. The initial lake bottom sludge and anammox sludge contained a diverse range of prokaryotic microorganisms, some of which were eliminated due to changing environmental conditions and interspecies competition.

**Table 2:** Alpha diversity analysis of microbial communities.

Sample ID	Feature	ACE	Chao1	Shannon	Simpson	Coverage
16sL_0_	2385	2408.588	2413.113	9.889	0.996	0.9988
16sA_0_	291	291.656	292	5.200	0.916	1.0
16sL_289_	862	880.491	879.705	7.837	0.985	0.9992
18sL_0_	453	454.004	453.526	6.060	0.947	0.9999
18sL_289_	247	247.159	247.000	4.147	0.853	1.0

#### 3.2.2 Microbial communities

**Fig. 3:**
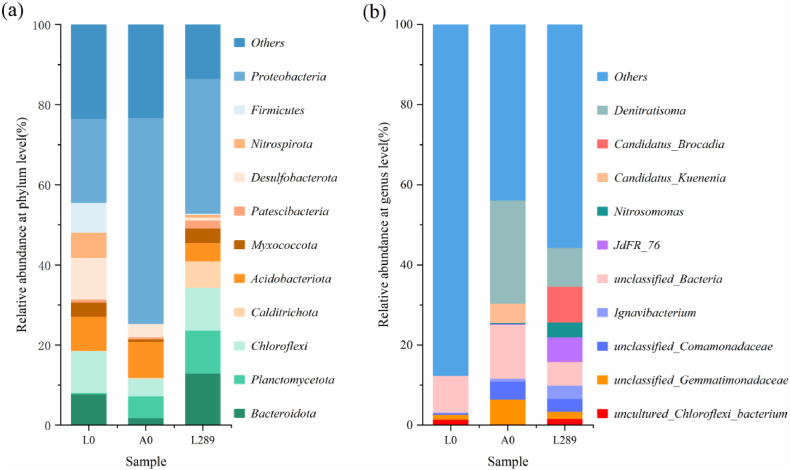
Relative abundance based on 16S rRNA gene: (a) at the phylum level. (b) at the genus level.

The changes in the relative abundance of species at the phylum level were shown in Fig. 3a. After 289 days of cultivation, significant increases in certain microorganisms at the phylum level were observed compared to the initial lake sediment and the inoculated anammox sludge. These included Planctomycetota, Bacteroidota, Calditrichota, and Patescibacteria. Meanwhile, Chloroflexi and Myxococcota showed minimal variation, whereas notable decreases at the phylum level were noted in Nitrospirota, Desulfobacterota, Firmicutes, and Acidobacteriota. Notably, Planctomycetota contains AnAOB, closely associated with nitrogen removal capacity. The initial lake bottom sludge exhibited a high proportion of Proteobacteria (20.95 %) and Nitrospirota (6.35 %). This microbial composition supports the hypothesis that nitrification occurred in the reactor during Stage I. It's worth mentioning that a majority of denitrifying bacteria belong to Proteobacteria, which partially contribute to NO_3_^−^-N removal. Nitrospirota were effectively inhibited, resulting in a decrease in their relative abundance from 6.35 % to 0.66 %. Nitrospirota may have been inhibited due to substrate deficiency resulting from the gradual decrease in artificially supplemented NO_2_^−^-N. NOB are known to be significantly inhibited under conditions of low dissolved oxygen concentration [17]. The micro-aerobic environment in the system may have contributed to the decrease in the relative abundance of Nitrospirota. Additionally, high pH (7.95 ± 0.40) and medium-high ammonia nitrogen concentration (100 mg/L) were also co-factors that could have contributed to partial nitrification [18].

At the genus level (Fig. 3b), denitrifying microorganisms in the inoculated anammox sludge and their relative abundances were *Denitratisoma* (25.71 %), *Candidatus Kuenenia* (4.83 %) and *Nitrosomonas* (0.30 %). In coupled systems, the predominant microorganisms included *Denitratisoma* (9.70 %), *Candidatus Brocadia* (8.87 %) and *Nitrosomonas* (3.74 %). *Denitratisoma* was inhibited, leading to a decrease in its relative abundance from 25.71 % to 9.70 %. Plant roots in the lake bottom sludge provided naturally occurring organic matter, which served as a carbon source for denitrifying bacteria at the beginning of the experiment. Subsequently, microalgae were used as a carbon source to facilitate endogenous denitrification. Additionally, the addition of microalgae promoted the production of extracellular polymeric substances, creating anaerobic zones within the granules and facilitating the development of denitrifying bacteria [19,20]. This may explain why *Denitratisoma* is not fully suppressed in the system. Notably, in the anammox sludge initially used for inoculation, *Candidatus Kuenenia* was the only anammox bacterium present, with a relative abundance of 4.83 %. However, by the 313th day, *Candidatus Brocadia* had become the dominant species, comprising 8.87 % of the community, while the relative abundance of *Candidatus Kuenenia* had decreased to 0.64 %. On one hand, a recent study showed that *Candidatus Brocadia* had adapted to light exposure, with symbiotic relationships within the community supporting this adaptation [21]. On the other hand, microalgae aggregated and grew at the periphery of the granular sludge due to phototropism, reducing the light intensity within the sludge. This environment may have allowed *Candidatus Brocadia* to gradually acclimate to the light conditions (Fig. 5a). The AOB was successfully enriched by reducing the influent NO_2_^−^-N/NH_4_^+^-N ratio and HRT. The relative abundance of *Nitrosomonas* increased from 0.30 % to 3.43 %. The experimental results indicated that NH_4_^+^-N was mainly utilized by anammox bacteria *Candidatus Brocadia* and nitrosobacteria *Nitrosomonas*.

### 3.3 Eukaryotic microbial communities

#### 3.3.1 Alpha diversity analysis

The alpha diversity index statistics for the microbial community were presented in Table 2. 18sL_0_ represented the initial lake bottom sludge, and 18sL_289_ represented the sludge sample collected on day 289. The 18S rRNA gene was not used to identify the anaerobic sludge because it originated from a completely anaerobic, light-free environment devoid of algae or microfauna. According to the ACE index and Chao1 index, the species richness of microorganisms in the reactor was lower than that of the initially inoculated lake bottom sludge. Similarly, the Shannon and Simpson index values indicated that the diversity of the microbial community in the reactor was reduced compared to the initial sludge. The initially inoculated lake bottom sludge contained abundant microalgae, protozoa, and metazoans. After a long period of experimentation, robust microalgae adapted to the experimental conditions were enriched.

#### 3.3.2 Microbial communities

**Fig. 4:**
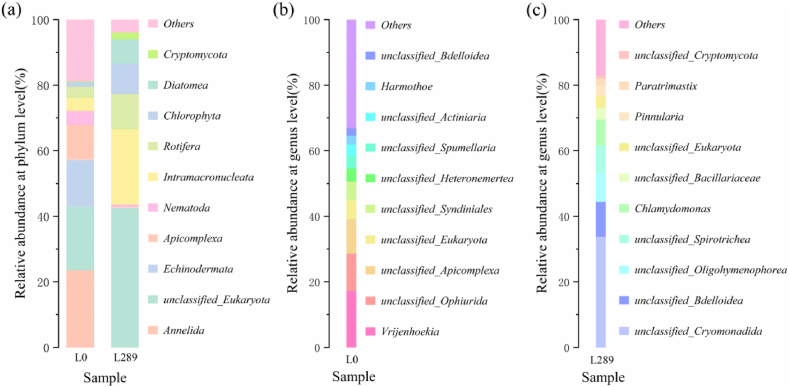
Relative abundance based on 18S rRNA gene: (a) at the phylum level. (b) sample L_0_ at the genus level. (c) sample L_289_ at the genus level.

At the phylum level, the results (Fig. 4a) indicated significant changes in the ALGAMMOX system compared to the initial lake bottom sludge, including an enrichment of algae, an increased relative abundance of Intramacronucleata among protists, and a higher relative abundance of Rotifera among metazoans. By the end of the experiment, the relative abundance of Chlorophyta had increased from 1.13 % to 9.26 %, Diatomea had increased from 0.26 % to 7.50 %, while Intramacronucleata and Rotifera had increased from 4.00 % to 3.40 %–23.00 % and 10.70 %, respectively. Chlorophyta, a major phylum of microalgae, includes commonly used species in wastewater treatment such as Chlorella and Chlamydomonas. The feasibility of nitrogen removal by Chlorophyta has been demonstrated since the 1980s [22]. Diatoms are crucial for biological carbon sequestration, contributing to carbon deposition through cellular sedimentation [23]. In denitrification systems, Diatoms play a vital role in providing carbon sources for anaerobic heterotrophic bacteria. When complex nitrogen sources are available, Diatoms tend to preferentially take up ammonia nitrogen over nitrate nitrogen [24].

**Fig. 5:**
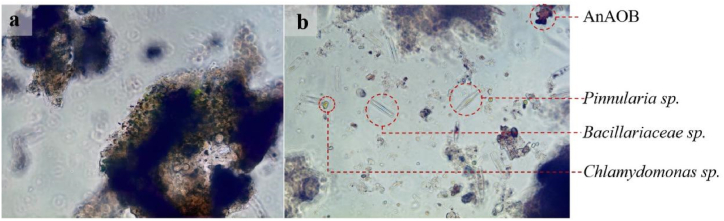
(a) Algal-bacterial consortium. (b) Some microorganisms in ALGAMMOX.

In terms of the relative abundance of species at the genus level (Fig. 4b and c), the dominant algal genera in the initial lake bottom sludge were *unclassified Syndiniales* and *unclassified Actiniaria*, with relative abundances of 5.67 % and 3.43 %, respectively. Throughout the incubation period, algae adapted to indoor natural light conditions and higher nitrite concentrations became enriched. The most prominent genus was *Chlamydomonas*, with a relative abundance of 7.99 %, followed by *unclassified Bacillariaceae* at 3.64 % and *Pinnularia* at 3.13 %. In terms of protozoans, the relative abundance of *unclassified Cryomonadida* in ALGAMMOX increased from 0.20 % to 33.85 % compared to the lake bottom sludge. Similarly, the relative abundances of *unclassified Oligohymenophorea* and *unclassified Spirotrichea* increased from their initial values of 0.12 % and 0.01 %–8.80 % and 8.31 %, respectively. Compared to the lake bottom sludge, the relative abundance of *unclassified Bdelloidea*, a member of metazoans in ALGAMMOX, increased from 2.27 % to 10.71 %. Various species of *Oligohymenophorea* exhibit distinct functions, with many Oligochaete species primarily feeding on planktonic algae [25]. A stable microfood web seems to have been established within this system, where protozoa consume microalgae and bacteria, and also serve as prey for microsecondary consumers. The sludge volume in the ALGAMMOX system did not notably increase. Microalgae growth remained within reasonable limits dictated by reactor characteristics, rather than excessive reproduction. Additionally, protozoa and metazoans likely consumed some of the microalgae.

Among the robust algae identified in the lake bottom sludge, *Chlamydomonas* stood out. *Chlamydomonas* assimilated nitrogen through various pathways, including both inorganic nitrogen (NO_3_^−^-N, NO_2_^−^-N, and NH_4_^+^-N) and organic nitrogen (urea, amino acids, and purines) [26]. Furthermore, *Chlamydomonas* was suggested to have participated in denitrification and ammonification processes [27]. Under nitrogen-starvation conditions, *Chlamydomonas reinhardtii* utilized excess carbon for fatty acid biosynthesis, accompanied by a significant increase in the expression of genes regulating lipid biosynthesis [23]. These factors likely contributed to the prominence of *Chlamydomonas* in the ALGAMMOX system. According to the results, *Chlamydomonas* appeared to be highly compatible with ALGAMMOX system. In addition, research has explored the potential of *Chlamydomonas* in biotechnology, including biohydrogen production, due to its resource recovery potential [28]. While *Chlamydomonas* seems well-suited for the algal anaerobic ammonia oxidation system, further mechanistic studies are necessary to substantiate this observation.

Fig. 5 depicted the algal-bacterial consortium and microorganisms in ALGAMMOX on day 289. In Fig. 5a, red anammox granular sludge was observed within the algal-bacterial consortium, surrounded by a ring of green, rounded *Chlamydomonas*. Fig. 5b provided a clearer image of microalgae within the activated sludge. The extracellular polymeric substances produced by microalgae and bacteria were likely crucial components of the flocculating material in the algal-bacterial consortium [29]. Observations showed that the effluent clarified due to the tightly bound algal-bacterial consortium and its good settling performance.

**Fig. 6:**
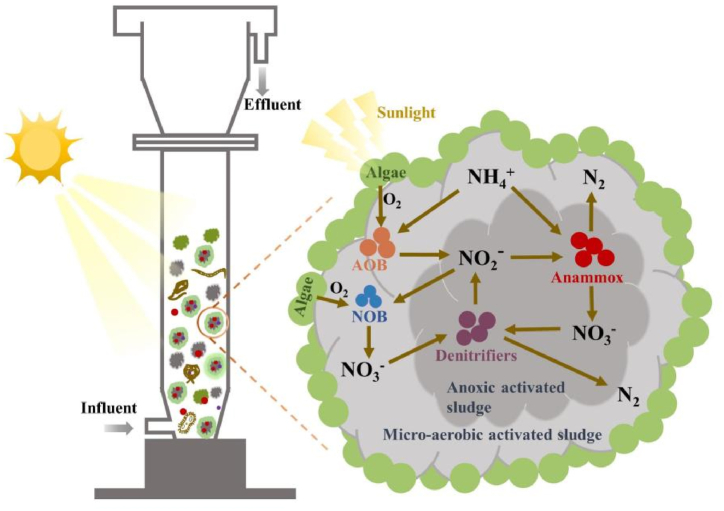
Schematic representation of the interactions between functional microorganisms of the algal-bacterial consortium.

Based on the observed structure of algal-bacterial consortium, prokaryotic microbial community analysis and eukaryotic microbial community analysis, a possible interaction principle between functional microorganisms in the algal-bacterial consortium was proposed (Fig. 6). The algal-bacterial consortium was composed of a microalgae layer, a micro-aerobic activated sludge layer, and an anaerobic activated sludge layer from the outside to the inside. Microalgae absorbed natural light to produce oxygen through photosynthesis. On one hand, oxygen was utilized by AOB in the micro-aerobic activated sludge to convert some of the NH_4_^+^ to NO_2_^−^. On the other hand, oxygen was also utilized by the NOB in the micro-aerobic activated sludge to convert NO_2_^−^ to NO_3_^−^. The AnAOB in the anaerobic activated sludge converted NH_4_^+^ and NO_2_^−^ into N_2_ and NO_3_^−^. Denitrifying bacteria in the anaerobic activated sludge reduced the NO_3_^−^ produced by NOB and AnAOB to N_2_, and the intermediate product NO_2_^−^ produced during the reduction process was also utilized by NOB and AnAOB.

## 4 Conclusion

The ALGAMMOX reactor was successfully initiated using lake bottom sludge and anammox sludge. The dominant bacterial microorganisms, *Denitratisoma* (9.70 %), *Candidatus Brocadia* (8.87 %), and *Nitrosomonas* (3.74 %), played a pivotal role in nitrogen removal. On day 289, the enrichment of microalgae in the ALGAMMOX system was evident, with *Chlamydomonas* being the most abundant species at 7.99 %, followed by *Bacillariaceae* at 3.64 %, and *Pinnularia* at 3.13 %. The peak nitrogen removal rate in the system was only 4.73 mg L^−1^ h^−1^. Future research activities should include strategies to enhance nitrogen removal rates and improve system stability.

## Data availability statement

All available data generated by experiments mentioned in this article are included in the manuscript. Raw datasets used and/or analyzed during the current study are available from the corresponding authors upon reasonable request.

## Ethics approval and consent to participate

Not applicable.

## CRediT authorship contribution statement

**Xiangyin Liu:** Writing – original draft, Methodology, Investigation, Formal analysis, Data curation, Conceptualization. **Jiannv Chen:** Writing – review & editing, Supervision. **Tiansheng Lu:** Writing – review & editing. **Yujie Qin:** Writing – review & editing, Project administration, Funding acquisition.

## Declaration of competing interest

The authors declare that they have no known competing financial interests or personal relationships that could have appeared to influence the work reported in this paper.

## References

[1] Gong S., Qin Y., Zheng S., Lu T., Yang X., Zeng M., Zhou H., Chen J., Huang W. (2023). The rapid start-up of canon process through adding partial nitration sludge to anammox system. J. Environ. Manag..

[2] Yang M., Xie K., Ma C., Yu S., Ma J., Yu Z., Chen X., Gong Z. (2022). Achieving partial nitrification-anammox process dependent on microalgal-bacterial consortia in a photosequencing batch reactor. Front. Bioeng. Biotechnol..

[3] Chen J., Liu X., Lu T., Liu W., Zheng Z., Chen W., Yang C., Qin Y. (2024). The coupling of anammox with microalgae-bacteria symbiosis: nitrogen removal performance and microbial community. Water Res..

[4] Zhang C., Li S., Ho S. (2021). Converting nitrogen and phosphorus wastewater into bioenergy using microalgae-bacteria consortia: a critical review. Bioresour. Technol..

[5] Li S., Zhang C., Li F., Ren N., Ho S. (2023). Recent advances of algae-bacteria consortia in aquatic remediation. Crit. Rev. Environ. Sci. Technol..

[6] Manser N.D., Wang M., Ergas S.J., Mihelcic J.R., Mulder A., van de Vossenberg J., van Lier J.B., van der Steen P. (2016). Biological nitrogen removal in a photosequencing batch reactor with an algal-nitrifying bacterial consortium and anammox granules. Environ. Sci. Technol. Lett..

[7] Akizuki S., Kishi M., Cuevas-Rodríguez G., Toda T. (2020). Effects of different light conditions on ammonium removal in a consortium of microalgae and partial nitrifying granules. Water Res..

[8] Wang L., Qiu S., Guo J., Ge S. (2021). Light irradiation enables rapid start-up of nitritation through suppressing nxrb gene expression and stimulating ammoniaoxidizing bacteria. Environ. Sci. Technol..

[9] Chu Z., Huang D., Huang X., He J., Chen L., Wang J., Rong H. (2023). Achieving robust mainstream nitritation by implementing light irradiation: long-term performance and microbial dynamics. Bioresour. Technol..

[10] Mukarunyana B., van de Vossenberg J., van Lier J.B., van der Steen P. (2018). Photo-oxygenation for nitritation and the effect of dissolved oxygen concentrations on anaerobic ammonium oxidation. Sci. Total Environ..

[11] Singh S.P., Singh P. (2015). Effect of temperature and light on the growth of algae species: a review. Renew. Sustain. Energy Rev..

[12] Liu J., Wu Y., Wu C., Muylaert K., Vyverman W., Yu H., Muñoz R., Rittmann B. (2017). Advanced nutrient removal from surface water by a consortium of attached microalgae and bacteria: a review. Bioresour. Technol..

[13] Shangguan H., Liu J., Zhu Y., Tong Z., Wu Y. (2015). Start-up of a spiral periphyton bioreactor (spr) for removal of cod and the characteristics of the associated microbial community. Bioresour. Technol..

[14] Qin Y., Han B., Cao Y., Wang T. (2017). Impact of substrate concentration on anammox-ubf reactors start-up. Bioresour. Technol..

[15] Wu C., Qin Y., Yang L., Liu Z., Chen B., Chen L. (2020). Effects of loading rates and n/s ratios in the sulfide-dependent autotrophic denitrification (sdad) and anammox coupling system. Bioresour. Technol..

[16] Wang L., Gu W., Liu Y., Liang P., Zhang X., Huang X. (2022). Challenges, solutions and prospects of mainstream anammox-based process for municipal wastewater treatment. Sci. Total Environ..

[17] Le L., Lee S., Bui X., Jahng D. (2020). Suppression of nitrite-oxidizing bacteria under the combined conditions of high free ammonia and low dissolved oxygen concentrations for mainstream partial nitritation. Environ. Technol. Innov..

[18] Gao D., Peng Y., Li B., Liang H. (2009). Shortcut nitrification–denitrification by real-time control strategies. Bioresour. Technol..

[19] Xiong W., Wang S., Jin Y., Wu Z., Liu D., Su H. (2023). Insights into nitrogen and phosphorus metabolic mechanisms of algal-bacterial aerobic granular sludge via metagenomics: performance, microbial community and functional genes. Bioresour. Technol..

[20] Jin Y., Zhan W., Wu R., Han Y., Yang S., Ding J., Ren N. (2023). Insight into the roles of microalgae on simultaneous nitrification and denitrification in microalgal-bacterial sequencing batch reactors: nitrogen removal, extracellular polymeric substances, and microbial communities. Bioresour. Technol..

[21] Kong L., Zheng R., Feng Y., Du W., Xie C., Gu Y., Liu S. (2023). Anammox bacteria adapt to long-term light irradiation in photogranules. Water Res..

[22] Tam N.F., Wong Y.S. (1989). Wastewater nutrient removal by chlorella pyrenoidosa and scenedesmus sp. Environ. Pollut..

[23] Field C.B., Behrenfeld M.J., Randerson J.T., Falkowski P. (1998). Primary production of the biosphere: integrating terrestrial and oceanic components. Science.

[24] Takabayashi M., Lew K., Johnson A., Marchi A.L., Dugdale R., Wilkerson F.P. (2006). The effect of nutrient availability and temperature on chain length of the diatom, skeletonema costatum. J. Plankton Res..

[25] Sommer U., Sommer F., Feuchtmayr H., Hansen T. (2004). The influence of mesozooplankton on phytoplankton nutrient limitation: a mesocosm study with northeast atlantic plankton. Protist.

[26] Fernandez E., Galvan A. (2008). Nitrate assimilation in chlamydomonas. Eukaryot. Cell.

[27] Bellido-Pedraza C.M., Calatrava V., Sanz-Luque E., Tejada-Jiménez M., Llamas Á., Plouviez M., Guieysse B., Fernández E., Galván A. (2020). Chlamydomonas reinhardtii, an algal model in the nitrogen cycle. Plants.

[28] Touloupakis E., Faraloni C., Silva Benavides A.M., Torzillo G. (2021). Recent achievements in microalgal photobiological hydrogen production. Energies.

[29] Kuo-Dahab W.C., Stauch-White K., Butler C.S., Gikonyo G.J., Carbajal-Gonzalez B., Ivanova A., Dolan S., Park C. (2018). Investigation of the fate and dynamics of extracellular polymeric substances (eps) during sludge-based photogranulation under hydrostatic conditions. Environ. Sci. Technol..

